# Androgen Reduces Mitochondrial Respiration in Mouse Brown Adipocytes: A Model for Disordered Energy Balance in Polycystic Ovary Syndrome

**DOI:** 10.3390/ijms22010243

**Published:** 2020-12-29

**Authors:** Avi Lerner, Drashti Kewada, Ayan Ahmed, Kate Hardy, Mark Christian, Stephen Franks

**Affiliations:** 1 Institute of Reproductive and Developmental Biology, Imperial College London, London SW7 2AZ, UK; a.lerner@imperial.ac.uk (A.L.); drashti.kewada@gmail.com (D.K.); aahmed0693@gmail.com (A.A.); kate.hardy@imperial.ac.uk (K.H.); 2 School of Science & Technology, Nottingham Trent University, Nottingham NG1 4FQ, UK; mark.christian@ntu.ac.uk

**Keywords:** PCOS, androgens, brown adipose tissue, mitochondrial respiration, UCP1

## Abstract

Polycystic ovary syndrome (PCOS) is a common endocrinopathy that is associated with an adverse metabolic profile including reduced postprandial thermogenesis. Although abnormalities in adipose tissue function have been widely reported in women with PCOS, less is known about direct effects of androgen on white and, particularly, brown adipocytes. The purpose of this study was to investigate the effect of the nonaromatizable androgen dihydrotestosterone (DHT) on (1) lipid accumulation and expression of adipogenic markers in immortalized mouse brown adipose cell lines (IMBATs), (2) mitochondrial respiration in IMBATs, (3) mitochondrial DNA content and gene expression, (4) expression of brown adipose tissue (BAT) markers and thermogenic activation. In addition, we profiled the relative levels of 38 adipokines secreted from BAT explants and looked at androgen effects on adipokine gene expression in both IMBATs and immortalized mouse white adipose (IMWATs) cell lines. Androgen treatment inhibited IMBAT differentiation in a dose-dependent manner, reduced markers of adipogenesis, and attenuated the β-adrenoceptor-stimulated increase in uncoupling protein-1 (UCP1) expression. In explants of mouse interscapular BAT, androgen reduced expression of UCP1, peroxisome proliferator-activated receptor-γ coactivator-1 (PCG-1) and Cidea. Significantly, as well as affecting genes involved in thermogenesis in BAT, androgen treatment reduced mitochondrial respiration in IMBATs, as measured by the Seahorse XF method. The results of this study suggest a role for excess androgen in inhibiting brown adipogenesis, attenuating the activation of thermogenesis and reducing mitochondrial respiration in BAT. Together, these data provide a plausible molecular mechanism that may contribute to reduced postprandial thermogenesis and the tendency to obesity in women with PCOS.

## 1. Introduction

Classically considered little more than a passive reservoir for energy storage, adipose tissue is now recognized as an active endocrine organ, involved in the regulation of metabolism and whole-body energy homeostasis. Adipocytes secrete a wide range of hormones and cytokines, collectively known as adipokines, that can modulate several physiological systems including regulation of appetite and satiety, insulin sensitivity, energy expenditure and reproductive function [1,2,3,4].

Adipose tissue exists in two main forms: white adipose tissue (WAT) and brown adipose tissue (BAT), and these differ in both morphology and physiology. WAT stores large amounts of energy in a unilocular lipid droplet, ready to be released as fatty acids for any future metabolic needs [5] whereas BAT is distinguished by its unique capacity to dissipate energy as heat in a process called non-shivering thermogenesis [6]. BAT is characterized by multiple small lipid droplets and abundant mitochondria and responds to cold or adrenergic stimuli by uncoupling oxidative phosphorylation from ATP synthesis to generate heat via the actions of uncoupling protein 1 (UCP1) [6]. Positron-emission tomography (PET) has confirmed the existence of functional BAT in the paracervical and supraclavicular regions of healthy, human adults [7]. Furthermore, it has become increasingly recognized that there is an inverse correlation between the amount of functional BAT and BMI, suggesting that its energy dissipating function may play a role in the development of obesity [8].

Adiposity is emerging as an important factor in causing or, at least exacerbating, common and complex disorders such as type-2 diabetes and polycystic ovary syndrome (PCOS). PCOS is the most common endocrinopathy in women, affecting between 5% and 10% of females of reproductive age [9]. It is characterized by hyperandrogenemia, is the principal cause of menstrual irregularity and anovulatory infertility and is associated with an adverse metabolic profile including obesity and insulin resistance. It is a significant risk factor for development of type-2 diabetes [10]. The prevalence of overweight or obesity in women with PCOS is between 40% and 90% and there is a strong association between adiposity and the severity of the clinical, biochemical and metabolic features of PCOS [11]. Furthermore, even modest weight loss of 5% of body weight has been shown to result in significant improvements in both symptoms of hyperandrogenism and ovulatory function [12].

While a clear link between increased adiposity and the severity of PCOS exists, the relationship between hyperandrogenemia and adipose tissue is less clear. There is evidence that increased adiposity directly increases androgen synthesis, partly due to accompanying hyperinsulinemia but also as a result of an effect of adipokines on ovarian steroidogenesis [10,13]. Comim et al. showed that, using cultured theca cells, the adipokines visfatin and leptin increased production of androstenedione while, in contrast (but in line with its anti-obesity effects), adiponectin suppressed production of androstenedione and steroidogenic enzymes [13]. Adipocyte dysfunction has been previously reported in women with PCOS with hyperandrogenism thought to be a driving factor. Increased adipocyte diameter and depot-specific changes in catecholamine-mediated lipolysis have also been reported in women with PCOS [14,15]. Differentiation of 3T3-L1 cells into adipocytes is inhibited by testosterone and androgens inhibit differentiation of human preadipocytes and reduce lipid metabolism in human adipose tissue explants [16,17]. AR knockout mice, in contrast, show increased adipogenesis and adipocyte size [18]. Finally, using transcriptomics, recent studies have looked at the global response to androgens in mice treated with dihydrotestosterone (DHT) [19] and demonstrated that, in agreement with previous studies, genes involved in adipocyte differentiation, adipogenesis and energy expenditure are modulated by androgen treatment.

The close association of PCOS with obesity and metabolic dysfunction suggests a role for adipokines as a link between inherent metabolic and reproductive abnormalities. A few studies have looked at a small number of adipokines in women with and without PCOS. The overarching trend is for adipokine levels to be raised in serum of women with PCOS and this is the case for resistin, leptin and visfatin [20]. In contrast, adiponectin levels are reduced in women with PCOS and this is supported by an in-vitro study by Xu et al., revealing that testosterone reduces the secretion of high molecular-weight adiponectin in visceral adipose tissue [21]. Although abnormal serum levels of adipokines in PCOS have been reported, little is known about the direct effects of androgens on adipokine expression in adipose tissue itself [22].

Importantly, there is evidence that androgens have an inhibitory action on BAT function and that this effect has implications for the development and manifestation of PCOS. Firstly, there are sex differences in energy expenditure, with female rodents being more efficient than males in the adaptive production of heat in response to feeding or postprandial thermogenesis [23]. Support for this being an androgen-mediated phenomenon is provided from a study of neonatally androgenized female mice which have been shown to have decreased brown adipose mass and reduced energy expenditure [24]. Mice that overexpress the androgen receptor have less BAT. In a prenatally androgenized sheep model of PCOS, postprandial thermogenesis is also suppressed [25]. Furthermore, testosterone has been shown to reduce UCP1 and mitochondrial biogenesis in rat BAT in-vitro and to inhibit the transcription of PGC1a, a key element in UCP1 expression and mitochondrial biogenesis [26,27]. The likely relevance of BAT function to PCOS is further illustrated by studies which indicate that stimulation of BAT activity [28] or transplantation of BAT to rodent models of PCOS can improve reproductive and metabolic function [22].

In the present study, we evaluated the expression of the androgen receptor (AR) protein in immortalized, mouse brown adipose cell lines (IMBATs), examining expression in both preadipocytes and fully differentiated adipocytes. We then investigated the effect of the nonaromatizable androgen DHT on lipid accumulation and expression of adipogenic markers in differentiated IMBATs. We examined the effect of androgen exposure on interscapular BAT explants by measuring gene expression of key BAT markers including uncoupling protein-1 (UCP1) and peroxisome proliferator-activated receptor-γ coactivator-1 (PCG-1). To understand BAT function in more detail we profiled the relative levels of 38 adipokines secreted from BAT explants. The effects of DHT on gene expression of a panel of 25 adipokines in IMBATs were compared with the profile obtained after similar treatment of IMWATs.

Finally, we investigated the effect of androgen on BAT energy expenditure by pretreating brown adipocytes with DHT and then measuring the β-adrenoceptor-stimulated increase in UCP1. We went on to examine the effect of androgen on mitochondrial respiration in BAT. These studies were designed to improve our understanding of androgen interactions with BAT function and how this could contribute to a disordered energy balance in PCOS.

## 2. Results

### 2.1. Androgen Receptor Protein Is Expressed in IMBAT Preadipocytes and Adipocytes

**Figure 1:**
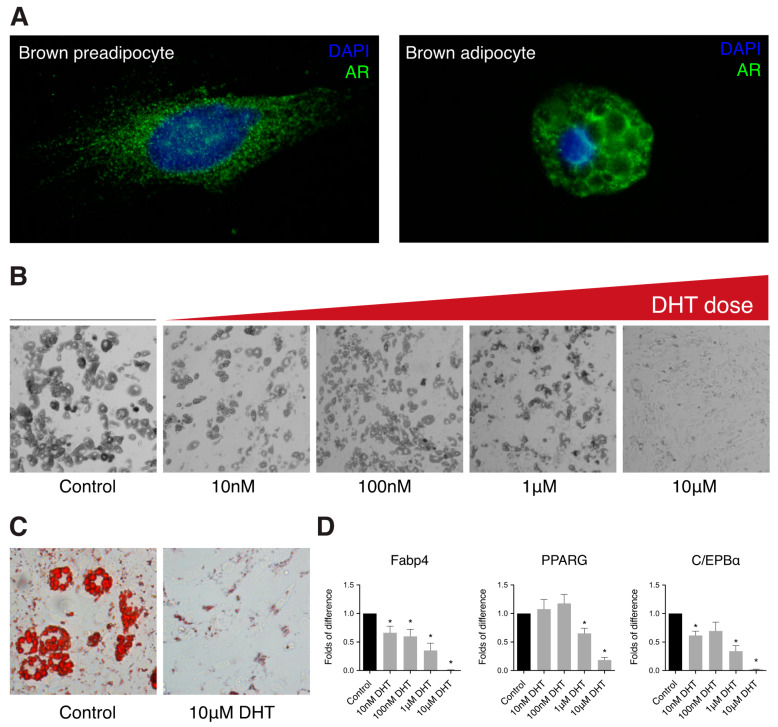
Dihydrotestosterone (DHT) treatment reduces brown adipogenesis. (**A**) Representative images showing presence of the androgen receptor protein in brown preadipocytes and adipocytes. Blue—nuclear stain DAPI, green—androgen receptor. (**B**) Representative differential interference contrast (DIC) microscopy images showing a reduction of lipid accumulation and brown adipocyte differentiation with increasing dose of DHT. (**C**) Representative images showing a reduction in lipid droplet formation with DHT, measured by Oil Red O in control and the 10 μM DHT. (**D**) Relative mRNA expression of adipogenic markers in brown adipocytes differentiated for seven days in the presence of different doses of DHT (*t*-test, * *p* < 0.05. Data shown is mean (±SEM *n* = 4).

Androgen receptor protein was detected by immunohistochemistry in preadipocytes and differentiated IMBATs. Expression of AR protein, in cells that had not been exposed to DHT, was located predominantly in the cytoplasm of preadipocytes and in the cytoplasm surrounding the lipid droplets in the differentiated adipocyte, as well as the preadipocytes (Figure 1A).

### 2.2. DHT Inhibits IMBAT Preadipocyte Differentiation in a Dose-Dependent Manner

To examine the role of androgen signaling in brown adipocyte differentiation, IMBATs were differentiated in the presence or absence of graded concentrations of DHT for seven days. Increasing concentrations of DHT resulted in a dose-dependent reduction in adipocyte differentiation and lipid accumulation (Figure 1B,C). To complement these morphological studies, we investigated the effects of a range of DHT doses on gene expression during differentiation. Markers of adipogenesis were significantly downregulated in a dose-dependent manner including Fabp4 (*p* < 0.05), C/EBPα (*p* < 0.05) and PPARG (*p* < 0.05) (Figure 1D).

### 2.3. Effects of DHT on Mitochondrial Respiratory Function in IMBATs

**Figure 2:**
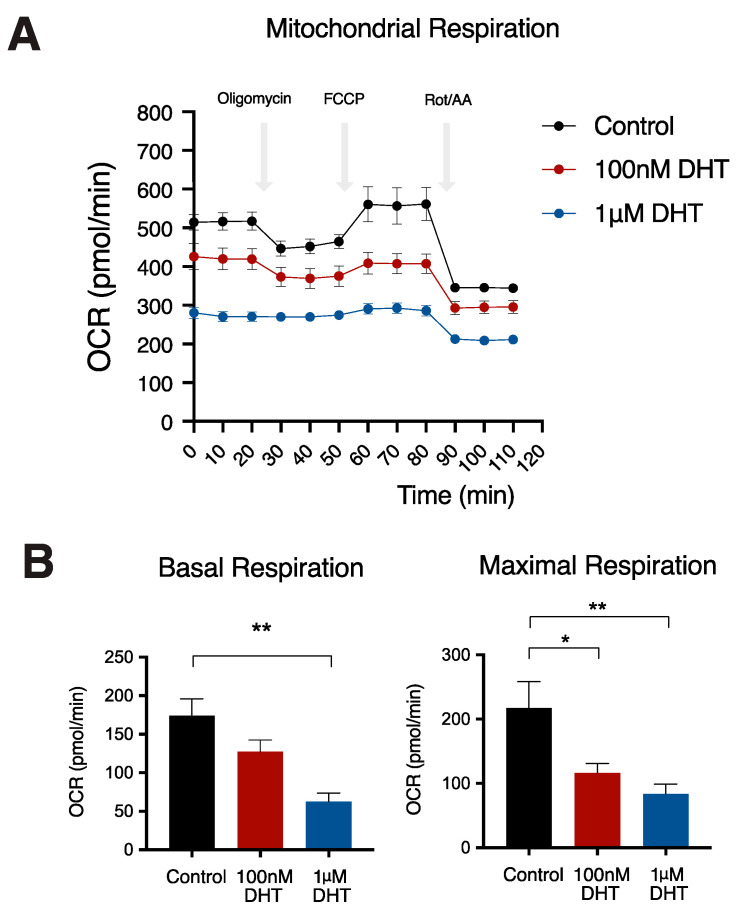
Reduced mitochondrial respiration in brown adipocytes treated with DHT. (**A**) Bioenergetic profile of brown adipocytes treated with control (black line), 100 nM DHT (red line) and 1μM DHT (blue line). (**B**) Oxygen consumption rate (OCR) shows reduction in both basal and maximal respiration in brown adipocytes treated with either 100 nM DHT (red bar) and 1μM DHT (blue bar). One-way ANOVA with Holm–Sidak’s multiple comparison test, * *p* < 0.05, ** *p* < 0.01. Data shown are mean (±) SEM, *n* = 6.

To explore the effect of DHT on mitochondrial respiration, we measured oxygen consumption rate (OCR) of IMBATs using a Seahorse XF analyzer. DHT treatment reduced the oxygen consumption rate (OCR) in IMBATs (Figure 2A). Basal mitochondrial respiration was reduced by 64.2% (*p* < 0.01) when the cells had been incubated in medium with 1μM DHT (Figure 2B). Maximal respiration was reduced by 9.68% (*p* < 0.05) and 26.9% (*p* < 0.01), respectively, when the cells were incubated in medium with 100 nM and 1μM DHT.

### 2.4. Effect of DHT on Brown Adipose Tissue Explants

**Figure 3:**
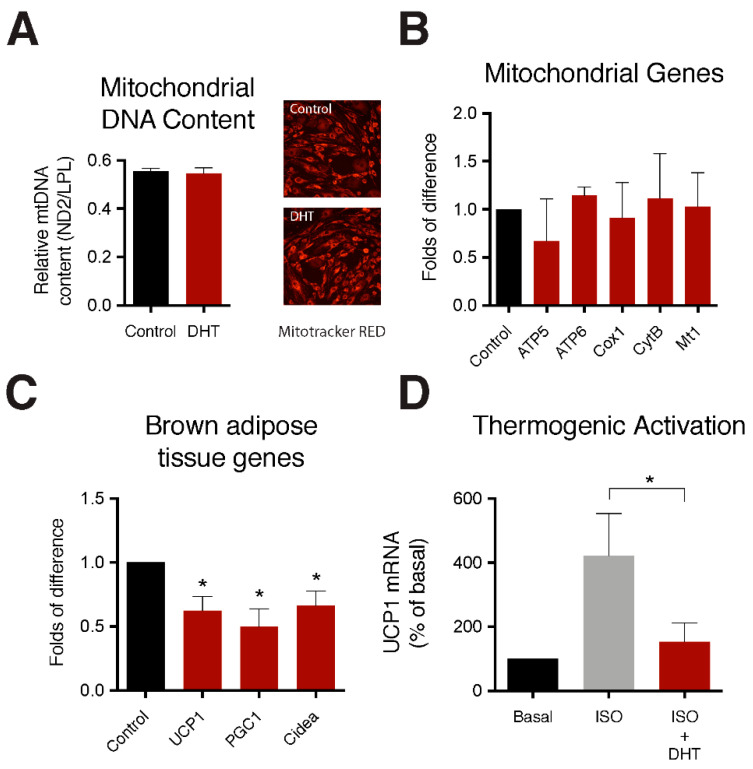
DHT treatment reduces UCP1 gene expression and activation of the thermogenic pathway. (**A**) Relative mitochondrial DNA content in brown adipose tissue treated with either control or 100 nM DHT. Representative images showing no change in mitochondrial number, observed by MitoTracker Red in control and the DHT treated brown adipocytes. (**B**) Relative mRNA expression of mitochondrial function genes in brown adipose tissue treated with either control or 100 nM DHT. (**C**) Relative mRNA expression of brown adipose markers in brown adipose tissue treated with either control or 100 nM DHT. (**D**) Thermogenic activation measured by UCP1 mRNA increase in brown adipocytes treated with either control, 1μM isoproterenol, or 1 μM isoproterenol co-treated with 100 nM of DHT. *t*-test, * *p* < 0.05. Data shown is mean (±) SEM *n* = 4).

To further understand the effect of androgens on markers of brown adipose we treated mouse interscapular BAT explants with 100 nM DHT for 24 h. Androgen treatment had no significant effect on mitochondrial DNA (mtDNA) content (Figure 3A) or mitochondrial gene expression (Figure 3B). However, androgen treatment caused a significant reduction in several key metabolic markers of brown adipose tissue including UCP1, PCG1 and Cidea (Figure 3C).

### 2.5. Effect of DHT on the Activation of the Thermogenic Gene Pathway

Fully differentiated IMBATs were treated with the β-adrenergic agonist isoproterenol to simulate activation of the thermogenic pathway. Cells were cultured with and without 100 nM DHT and expression levels of UCP1 mRNA were measured by qPCR. As expected, isoproterenol stimulated UCP1 expression. Androgen treatment significantly reduced the increase in UCP1 expression to almost baseline levels (Figure 3D).

### 2.6. BAT Is an Active Endocrine Organ and Secretes an Array of Adipokines

**Figure 4:**
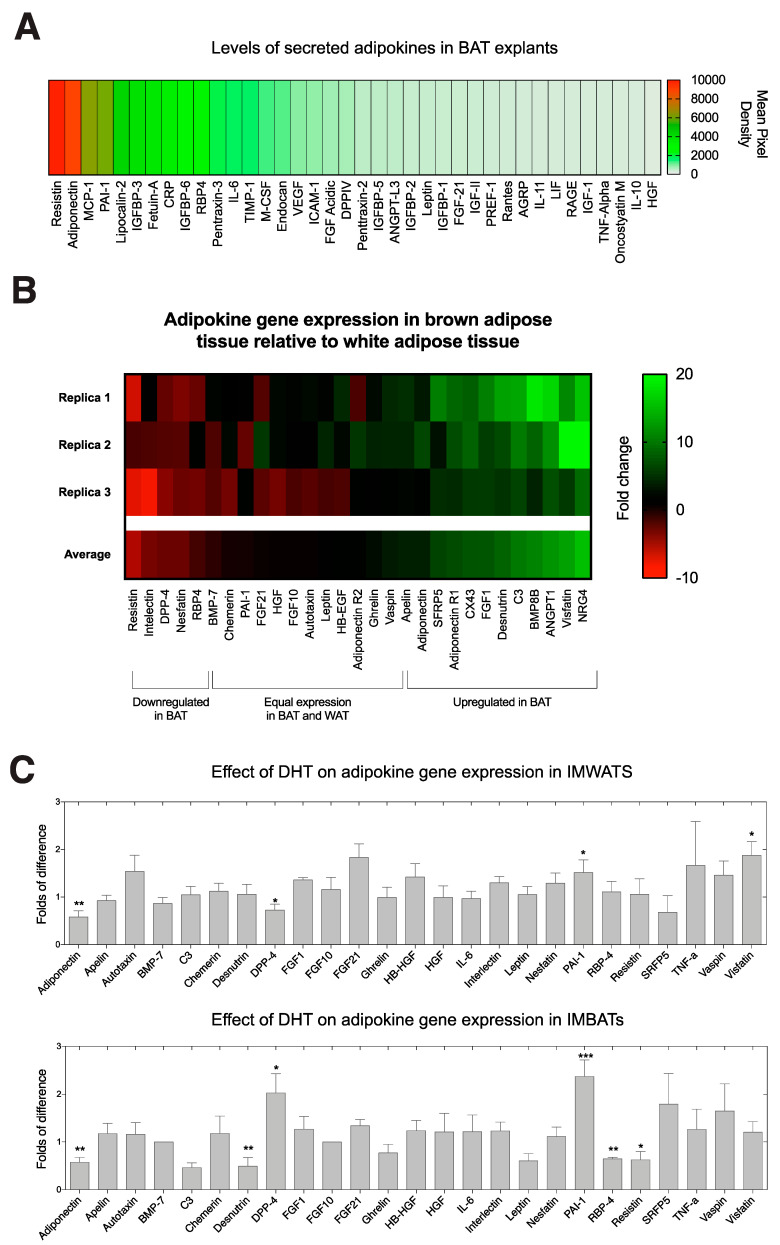
(**A**) Heat-map produced from proteome profiler adipokine arrays used to profile the levels of secreted adipokines from interscapular BAT explants cultured in control medium for 24 h. Red indicates adipokines secreted at high levels and green indicates adipokines secreted at lower levels from brown adipose tissue (*n* = 4). (**B**) Heat-map showing adipokine gene expression in BAT relative to WAT. On the scale bar, red indicates genes expressed at lower levels in BAT and green indicates genes expressed at higher levels in BAT, black represents genes that are expressed equally in BAT and WAT. (**C**) Relative mRNA expression of adipokine screen in IMWATs and IMBATs treated with either control of 10 μM DHT. *t*-test, * *p* < 0.05, ** *p* < 0.01, *** *p* < 0.005. Data shown are mean (±) SEM, *n* = 6–8.

To gain a better understanding of the adipokines secreted by intact BAT we used proteome profiler adipokine arrays to profile the relative amounts of 38 adipokines secreted from BAT explants over 24 h. Interestingly, BAT secreted high levels of several adipokines such as adiponectin and resistin that have been shown to be altered in women with PCOS or by high levels of androgen [20,21,29,30] (Figure 4A).

### 2.7. Explants of Brown Adipose Tissue Have Distinct Adipokine Gene Expression Profiles Compared to White Adipose Tissue

Using qPCR data, we compared the gene expression levels of adipokines in mouse WAT and BAT explants. Four adipokines were expressed at lower levels in BAT than in WAT. Approximately 15 adipokines shared similar levels of expression in BAT compared to WAT. However, 11 adipokines were expressed at higher levels in BAT than WAT (Figure 4B).

### 2.8. Effect of DHT on Adipokine Gene Expression in Differentiated IMWATs and IMBATs

To further characterize the response of brown adipocytes to androgen we looked at the effects on adipokine gene expression after treating fully differentiated IMBATs with DHT and examined changes in gene expression by qPCR for a panel of 25 adipokines. We compared the panel of expressed genes with that obtained after similar treatment of IMWATs. DHT treatment significantly altered the expression of several adipokines genes in IMBATs (Figure 4C). Of particular interest is expression of adiponectin that was significantly decreased by DHT (*p* < 0.01). Plasminogen activator inhibitor-1 (PAI-1) showed an increase (*p* < 0.001), C3, an immune marker was significantly decreased (*p* < 0.01) and resistin, an adipokine involved in obesity and Type-2 diabetes, was also significantly decreased (*p* < 0.05). In IMWATs there was a similar decrease in adiponectin expression (*p* < 0.01) and an increase in PAI-1 expression (*p* < 0.05). However, DPP-4 was downregulated in IMWATs (*p* < 0.05) but upregulated in IMBATs (*p* < 0.05). Visfatin was the only adipokine to be modulated solely in IMWATs and was significant upregulated (*p* < 0.05). Interestingly the magnitude of response to androgen treatment tended to be greater in IMBATs than in IMWATs (Figure 4C).

## 3. Discussions

In this study, we investigated the role of the nonaromatizable androgen DHT in a well characterized, immortalized mouse brown adipocyte cell line and in explants of mouse brown adipose tissue. The importance of adipose tissue in the pathophysiology of PCOS is well established but studies to date have mainly focused on the relevance of white adipose depots and the possible role of androgens in WAT dysfunction [17,31,32]. Interest in brown adipose tissues has been growing following the discovery of significant depots of metabolically active brown adipose tissue in healthy adult humans [7,33]. Brown adipose tissue was first described in small mammals and infants as an adaptation to defend against the cold [6,34]. The abundance of BAT lessens with increasing age and was thought to be completely lost by adulthood. BAT is the major site of adaptive non-shivering thermogenesis, both during cold exposure and after meals in so called postprandial thermogenesis. The inverse relationship between the BAT activity and body fatness suggests that BAT, through increasing energy expenditure, is protective against body fat accumulation [8,35]. This has led to BAT being viewed as a promising therapeutic target for combating human obesity and related metabolic disorders [36,37]. Studies in both rodents and humans have shown that BAT is activated on food intake [38,39,40]. Furthermore, postprandial thermogenesis is higher in individuals with higher BAT activities and it has been reported that polymorphisms in the UCP1 gene can negatively affect PPT after a high-fat meal [37,41]. Robinson and colleagues showed that while resting energy expenditure was similar in women with and without PCOS, postprandial thermogenesis was reduced in both obese and lean women with PCOS [42]. This decreased postprandial thermogenesis may predispose women with PCOS to weight gain and help to explain the increased prevalence of obesity in women with PCOS.

Our findings provide further evidence of androgens modulating mitochondrial respiration in adipose tissue and may help elucidate the molecular mechanisms underpinning disordered energy balance in women with PCOS. We show that brown preadipocytes and adipocytes contain androgen receptor and that brown adipogenesis is inhibited in a dose-dependent manner by androgen treatment. This can be clearly visualized through both a reduction in lipid droplets and key adipogenic markers. Androgen signaling may also play a direct role in the normal physiology of brown adipose tissue. We show that IMBATs cultured in the presence of DHT have significantly reduced mitochondrial respiration. These mitochondrial defects are likely to contribute to reduced energy expenditure and non-shivering thermogenesis.

Furthermore, DHT treatment appeared to have a “whitening” effect on interscapular BAT explants treated with androgens, as seen by a reduction of key markers of BAT including UCP1, PGC1 and Cidea. Although UCP1 is a major driving force in non-shivering thermogenesis, a number of other factors including mitochondrial density and biogenesis are required for effective thermogenesis and energy expenditure [43]. Our results show that expression of PGC-1α, a critical regulator of mitochondrial biogenesis was downregulated by androgen. While there was no effect of androgen exposure on either mitochondrial gene expression or mitochondrial DNA content, the observed reduction in mitochondrial respiration shows that mitochondrial function is significantly impaired by androgen.

From a clinical viewpoint, decreased mitochondrial respiratory activity is also implicated in the development of type-2 diabetes and is thought to be modulated, in part, by insulin resistance [44,45,46]. Insulin resistance plays a central role in the pathogenesis of both PCOS and type-2 diabetes and has a bearing on therapeutic strategy. Metformin has been widely used in management of PCOS and there are a growing number of clinical trials that suggest therapeutic benefits of novel inositol treatments [47,48]. There is also evidence that metformin treatment increases mitochondrial respiration, membrane potential, and ATP levels in hepatocytes [49].

Under normal physiological conditions, thermoregulation in mammals is coordinated and governed by the hypothalamus via the activation of the sympathetic nervous system. We wanted to investigate the effect of excess androgens on the activation of the thermogenic pathway. To this end, we exposed IMBATs to the β-adrenergic agonist, isoproterenol, in order to activate the thermogenic pathway and measured expression levels of UCP1, a well-accepted marker of thermogenesis activation [50,51]. We found that brown adipocytes pretreated with androgens showed reduced levels of UCP1 and hence reduced activation of the thermogenic pathway. This could provide one possible mechanism to understand reduced postprandial thermogenesis found in women with PCOS. In a similar fashion Barbato et al., recently showed that glutathione decrement drives the thermogenic program in adipose cells. However, adipocytes co-treated with isoproterenol and a glutathione ester showed blunted activation of the thermogenic program [51]. These results provide insight into the molecular mechanism that might underlie similar findings in a recently published study of a prenatally androgenized sheep model of PCOS. This study showed postprandial thermogenesis is also suppressed by androgens and was associated with a reduction of UCP1 in interscapular brown adipose tissue [25].

Finally, we characterized adipokine profiles in brown adipose tissue. Studies of animal models of PCOS suggest that exposure to excess androgen during or well before puberty increases adiposity and influences circulating levels of adipokines [52]. Several studies have suggested a role for adipokines as a link between the typical metabolic and reproductive abnormalities found in PCOS [13,20,53,54]. Our results also show that BAT, like WAT, is an active endocrine organ and secretes a range of adipokines, Adiponectin, resistin and PAI-1 are all secreted at high levels from BAT and production of these key adipokines is commonly disordered in PCOS. It is important to note that although BAT is an active endocrine organ and secretes a range of adipokines, BAT and WAT show distinctly different adipokine gene expression profiles. Furthermore, in a screen of adipokine abundance in androgen treated IMBATS, we observed a significant reduction in adiponectin (an “anti-obesity” adipokine) and resistin expression while PAI-1 was significantly increased. A similar effect was observed in IMWATs treated with androgen. Adiponectin expression was reduced and PAI-1 expression increased by DHT. The reduction of adiponectin is consistent with data showing that women with PCOS and androgenized mice have lower serum adiponectin levels than in controls and reduced adiponectin gene expression in subcutaneous and omental adipose depots has been demonstrated in women with PCOS [20,52,55,56]. We also show that there was a greater response in adipokine production to androgen treatment in brown adipocytes when compared to white adipocytes. While both IMWATs and IMBATs show a reduction in adiponectin after DHT treatment, IMBATs but not IMWATs also have reduced production of resistin, desnutrin/ATGL and RBP-4. Reduced levels of desnutrin/ATGL, the rate limiting lipase enzyme may impair the release of free fatty acids required for oxidation and activation of UCP1. The lower levels of BAT-associated genes support the notion of a “whitening” of brown adipocytes in response to androgen treatment. DPP-4, while downregulated in IMWATs is upregulated in IMBATs and would likely impact on insulin responses due to action on GLP-1 and incretin.

Although these studies focus on the nonaromatizable androgen DHT it is important to remember that aromatase activity may play a role in androgen and estrogen interactions with BAT in-vivo. In particular, estrogen is known to increase expression of UCP genes in rodent adipose tissue [57] and regulate thermogenesis in BAT via hypothalamic AMPK [58].

In conclusion, our study illustrates a novel role for androgen signaling in inhibiting mitochondrial respiration, brown adipogenesis and in “whitening” of brown adipose tissue. Androgen treatment attenuates the β-adrenoceptor-stimulated increase in UCP1 in IMBATs and provides a molecular explanation for the link between hyperandrogenemia and reduced thermogenic capacity. Together, these findings provide insight into the molecular mechanisms that underlie normal regulation of BAT but, in particular, highlight a potential role for androgen action on BAT function that may contribute to the pathophysiology of PCOS and associated disorders of energy balance.

## 4. Materials and Methods

### 4.1. IMBAT and IMWAT Preadipocyte Culture and Adipocyte Differentiation

Immortalized mouse brown (IMBATs) preadipocytes were cultured at 33 °C with 10% CO2 in DMEM/F12 1:1 (Invitrogen, Life Technologies, Paisley, Scotland), supplemented with 4.5 g/L glucose, 10% FBS (Sigma-Aldrich Co.; St. Louis, MO, USA), and 1% penicillin/streptomycin (BioWhittaker, Vervier, Belgium) as described previously [59]. IMBATs were differentiated in DMEM/F12 1:1 with 4.5 g/L glucose, 10% FBS, 1% penicillin/streptomycin supplemented with 170 nM insulin; 250 nM dexamethasone; 500 μM isobutylmethylxanthine; 125 nM indomethacin (Sigma) and 1 nM T3 (Sigma) at 37 °C with 5% CO2. After 48 h, the medium was replaced with fresh DMEM/F12 1:1, 4.5 g/L glucose, 10% FBS, 1% penicillin/streptomycin supplemented with 170 nM insulin and 1 nM triiodothyronine (T3) and cultured for a further 12 days until fully differentiated. IMWATs were differentiated in DMEM/F12 1:1 with 4.5 g/L glucose, 10% FBS, 1% penicillin/streptomycin supplemented with 170 nM insulin (Sigma); 250 nM dexamethasone (Sigma); 500 μM isobutylmethylxanthine (Sigma); 2.5 μM rosiglitazone (VWR, VWR Company, Darmstadt, Germany) at 37 °C with 5% CO2. After 48 h, the medium was replaced with DMEM/F12 1:1, 4.5 g/L glucose, 10% FBS, 1% penicillin/streptomycin supplemented with 170 nM insulin and cultured for a further 12 days until fully differentiated. Medium was changed every 2–3 days.

### 4.2. Immunohistochemistry

IMBAT preadipocytes and adipocytes were washed twice in PBS and then fixed in 4% paraformaldehyde for 15 min at 37 °C. Cells were permeabilized using ice-cold acetone at −20 °C for 5 min and then washed three times with PBS. Non-specific binding was blocked using 20% normal goat serum incubation for 1 h at room temperature. Cells were incubated overnight at 4 °C with a rabbit anti-androgen-receptor primary antibody at 1:300 dilution (Abcam, Cambridge, MA, USA), this concentration has been previously shown to work effectively in-house for mouse ovary immunohistochemistry. After several washes with PBS, the cells were incubated with an Alexa Fluro 488-conjugated secondary antibody for 1 h at room temperature. Cells were counterstained and preserved using Prolong Gold medium containing 4′6-diamidino-2-phenylindole (DAPI; Invitrogen) and examined under a fluorescent microscope (Nikon Eclipse TE300). Distribution of mitochondria was examined by live cell imaging using MitoTracker Red as per manufacturer’s instructions (Invitrogen, Carlsbad, CA, USA).

### 4.3. Effect of DHT on IMBAT Differentiation

To investigate the effect of androgens on brown adipocyte differentiation, IMBATs were differentiated using the protocol described above but this time in the presence of different doses of DHT (10 nM, 100 nM, 1 μM and 10 μM) or vehicle control (ethanol) for 7 days. Differential interference contrast (DIC) microscopy was used to judge the effect of different doses of DHT on IMBAT differentiation and lipid accumulation (Nikon Eclipse TE300, Nikon, Tokyo, Japan). Cells were collected to determine the effects of DHT on adipogenic markers. Cells were collected in QIAzol (QIAgen, Qiagen, Hilden, Germany) and stored at −80 °C until needed for gene expression studies.

### 4.4. Effect of DHT on IMBAT Oxygen Consumption Rate (OCR)

IMBATs were seeded at 2 × 104 cells/well in XFe24 microplate coated with 0.2% gelatin, the cells were differentiated as described above and treated with androgen for 48 h. The cells were washed twice, and medium was replaced with XF Assay medium containing 4.5 g/L glucose, 4.0 mM Glutamine and 1.0 mM Sodium pyruvate (pH adjusted to 7.35 ± 0.05 using 1 mol/L NaOH). The plates were placed in a 37 °C incubator without CO2 for one hour prior to the assay. OCR measurements were performed using the Seahorse Biosciences XFe Analyzer. All experiments were performed at 37 °C. After measurement of basal respiration, oligomycin (2.0 μM), FCCP (1.0 μM), rotenone/antimycin A (1.0 μM/1.0 μM) were added sequentially to measure ATP production, maximal respiratory, and non-mitochondrial respiration (NMR), respectively.

### 4.5. Brown Adipose Tissue Explants and DHT Treatment

C57BL/6 female mice were culled and the interscapular brown adipose tissue and inguinal white adipose tissue collected and processed immediately for quantitative, real time PCR (qPCR). For culture experiments, fresh brown adipose tissue was cut into 5–10 mg pieces and cultured in 12-well plates in 1 mL serum-free DMEM/F12, 1:1 supplemented with 1% penicillin/streptomycin at 37 °C with 5% CO2. BAT explants were treated with either 100 nM DHT or vehicle control (ethanol) for 24 h and collected for subsequent gene expression analysis by QPCR. Cultured medium was collected for adipokine array analysis. All animal studies were carried out according to UK Home Office guidelines. To investigate the effect of androgens on the activation of the thermogenic pathway, IMBATs were fully differentiated and were serum starved in DMEM/F12 1:1 with 4.5 g/L glucose, 1% penicillin/streptomycin and no FBS for 24 h. Cells were then treated with either 100 nM DHT or vehicle control (ethanol) for 24 h. After 19 h, all cells were treated with 1 μM isoproterenol (VWR) for 5 h. Cells were collected in QIAzol (QIAgen, Qiagen, Hilden, Germany) and UCP1 gene expression levels were measured.

### 4.6. RNA Extraction and Quantitative Real Time PCR

Samples were homogenised in QIAzol using a QIAShredder column (QIAgen, Qiagen, Hilden, Germany). Total RNA extraction used a method combining both QIAzol and QIAgen Mini RNA kits [60]. Purity and quantity of RNA was measured using a Nanodrop 1000 (NanoDrop Technologies, Wilmington, DE, USA). 1 μg of RNA was reversed transcribed using random hexamers and Superscript III according to manufacturer’s instructions (Invitrogen). QPCR was carried out in 384 well plates using POWER SYBR Green (Applied Biosystems, Foster City, CA, USA), according to the manufacturer’s instructions, on an Applied Biosystems 7900 HT instrument. Primers were designed with Primer3 [61] (available on request). Relative expression levels were determined using the delta delta Ct method [62]. Control samples were normalized to 1 and actin B was used as an internal reference gene and has previously been shown to be an appropriate and stable reference gene for adipose tissue [63,64,65]. All samples were analysed in triplicate and dissociation curves were used to ensure that a single product was formed. For mitochondrial DNA quantification, the ratio of mtDNA (ND2) to nuclear DNA (LPL) in adipocytes, which reflects the cellular mitochondrial number, was determined by qPCR. DNA was isolated from adipocytes according to the manufacturer’s protocol (Qiagen, Valencia, CA, USA).

### 4.7. Expression of Adipokine Genes in IMWATs and IMBATs after DHT Treatment

To investigate the response of adipokine genes to excess androgen treatment we fully differentiated IMWATs and IMBATs for 12 days. The cells were then serum starved in DMEM/F12 1:1 with 4.5 g/L glucose, 1% penicillin/streptomycin and no FBS for 24 h. Cells were then treated with either 10 μM DHT (Sigma) or vehicle control (ethanol) for 24 h. Cells were collected in QIAzol (QIAgen, Qiagen, Hilden, Germany) and stored at −80 °C until needed for adipokine gene expression analysis. A panel of 25 adipokines were selected based on their relevance to PCOS, especially those based on obesity and insulin resistance.

### 4.8. Secreted Adipokine Array

To further characterize brown adipose tissue in mice, we used the Proteome Profiler Mouse Adipokine Array Kit (R & D, Minneapolis, MN, USA) to measure the levels of secreted adipokines in BAT cultured media, using the manufacturer’s instructions. Proteome Profiler membranes were visualized using a ImageQuant LAS 4000 (Amersham, The Netherlands).

### 4.9. Statistical Analysis

Statistical analysis of qPCR data was carried out according to Schmittgen [62] using a two-tailed, unpaired Student *t*-test. A *p*-value of < 0.05 was considered statistically significant. Adjustment for multiple comparisons was made using the Benjamini–Hochberg method [66]. Data are presented graphically as mean (±SEM). Analysis was carried out using Prism 6.0 (GraphPad Software Inc., San Diego, CA, USA).
